# Inclusion of short-term care patients affects the perceived performance of specialists: a retrospective cohort study

**DOI:** 10.1186/s12913-015-0757-8

**Published:** 2015-03-14

**Authors:** Mark A Nyman, Rosa L Cabanela, Juliette T Liesinger, Paula J Santrach, James M Naessens

**Affiliations:** The Division of General Internal Medicine, Mayo Clinic, 200 1st Street SW, Rochester, MN USA; The Division of Health Care Policy & Research, Mayo Clinic, Rochester, MN USA; Department of Laboratory Medicine and Pathology (Dr. Santrach), Mayo Clinic, Rochester, MN USA

**Keywords:** Performance measurement, Specialty care, Short-term care, Long term care

## Abstract

**Background:**

Current publicly reported quality performance measures directly compare primary care to specialty care. Specialists see short-term patients referred due to poor control of their disease who then return to their local provider. Our study looked to determine if outcomes measured in short-term care patients differed from those in long-term care patients and what impact those differences may have on quality performance profiles for specialists.

**Methods:**

Retrospective cohort from a large academic medical Center. Performance was measured as “Optimal Care” - all or none attainment of goals. Patients with short-term care (<90 days contact) versus long-term care (>90 days contact) were evaluated for both specialty and primary care practices during the year 2008.

**Results:**

Patients with short-term care had significantly lower “Optimal Care”: 7.2% vs. 19.7% for optimal diabetes care in endocrinology and 41.3% vs. 53.1% for optimal ischemic vascular disease care in cardiology (p < 0.001). Combining short and long term care patients lowered overall perceived performance for the specialty practice.

**Conclusions:**

Factors other than quality affect the perceived performance of the specialty practice. Extending current primary care quality measurement to short-term specialty care patients without adjustment produces misleading results.

## Background

Since public reporting of performance data can motivate improvements in the delivery of high quality care [1-3], a move towards public performance reporting has occurred at the medical group and clinic level. One example, Minnesota Community Measurement (MNCM) was created in 2004 as a collaboration between payers, providers and employers, with the goal of publicly reporting quality data to accelerate the improvement of healthcare in the state of Minnesota [4]. Initial efforts focused on primary care clinics, providing longitudinal care, with documented improvement at the provider level [5] and at the state level with annual gains in many of these metrics [6]. More recently, public reporting has broadened to include specialty clinics, which are compared directly with primary care clinics using the same outcome metrics for diabetes and vascular disease. In the United States, most insurance plans allow patients to obtain services either through primary care, delivered by both general internal medicine and family medicine providers, or specialty care. However, specialty providers see two types of patients: those who they care for over the long-term and those who they care for over the short-term. Long term patients are generally those managed over time by the specialist, usually complex patients with challenging chronic disease control issues; whereas short-term patients tend to be those who have been referred for management recommendations and then return after their consultation to their primary care provider for continuing care. In a referral center, some of these short-term patients may still meet the multiple visit attribution criteria of existing measures. Current quality reporting does not adequately differentiate between short-term and long-term patients, but combines them together as one cohort for assessing specialist quality performance.

Following Donabedian’s model [7], quality can be assessed using structure, process or outcome measures. When comparing metrics across providers, measurement definitions become important, particularly for outcome measures. Patient characteristics have been shown to influence performance measures for both primary care and specialty care physicians [8,9]. Alternative methods of attributing a patient to a provider or clinic have been shown to impact performance measures and reported costs [10,11]. Studies comparing the performance of specialist care to primary care have shown contradictory results, though there have been methodological challenges [12]. Studies showing better performance for specialty care tend to evaluate patients receiving care over the long-term [9,13]. Laboratory and physiologic measures used as outcome measures during a short-term care episode can represent care provided prior to the measurement and not care received at the time of the specialist visit. For example, when adjusting medications, the American Diabetes Association (ADA) recommends an interval of 90 days prior to reassessment of the hemoglobin A1C [14]. So a hemoglobin A1C obtained during a short-term care episode (<90 days contact) would reflect the quality of prior care– not the quality of care delivered by the specialist. We found no studies evaluating the impact on the perceived performance of specialty practices when combining short-term care patients with long-term care patients.

We hypothesized short and long term care patients were significantly different and if combined together would cause a change in the perceived quality performance of specialists. We addressed these questions by comparing the differences between short and long term patients and comparing performance metrics between patients seen by primary care providers (general internal medicine and family practice) with those seen by specialist providers for two disease cohorts, diabetes and vascular disease, using the MNCM specifications for publically reported quality data.

## Methods

### Overview

To fully understand the impact on public measures, we created our cohorts and assessed outcome measures according to the current measurement specifications of the Minnesota State Quality Measures (as specified by MNCM). We compared the performance of specialists to primary care practitioners for two retrospective disease cohorts at a large academic medical center in Rochester, Minnesota: patients with diabetes and those with ischemic vascular disease (IVD). Cohorts were identified using institutional billing data. To be considered in the measurement population, per MNCM definitions [15], a patient needed to be seen at least twice during 2007 or 2008 (24 months) within the same primary care, cardiology or endocrinology clinic. At our institution, endocrinology and cardiology clinics are referral clinics staffed by Board-certified specialists, while primary care and general internal medicine clinics are staffed by board certified internal medicine and family medicine physicians. Following the public reporting specifications, we compared all patients with diabetes seen at our institution at least twice by an endocrinologist (and not a primary care provider) to all patients seen at least twice by a primary care provider. A similar comparison was made for the IVD cohort between cardiologists and primary care providers. For each patient, the most recent laboratory data (hemoglobin A1C and LDL cholesterol) and blood pressure values were extracted from the electronic medical record (EMR) as specified by MNCM. We defined short-term care as having the time between the initial and final visit within the two year window of less than 90 days, while long-term care was defined as visits with the provider occurring over more than 90 days. This timeframe was chosen based on clinical opinion of the minimal time necessary to see the outcomes of most chronic disease treatments and was consistent with the ADA recommended reassessment time of hemoglobin A1C. As this study was based on deidentified patient information used for public reporting, it was reviewed by the Mayo Clinic Institutional Review Board (09-008403) and deemed as exempt from further review.

### Study population

Patient cohorts were identified using the MNCM publicly reported quality metric definition applied to administrative billing data [15]. Patients, aged 18 to 75, had to have at least two visits for diabetes or vascular care within the years 2007 and 2008 with at least one office visit of any kind during 2008. Patients who died by December 31, 2008 were excluded. Diabetes and IVD visits were defined using ICD-9 codes as outlined in Table 1.

**Table 1:** **ICD-9 Diagnosis codes**

**Diabetes**	
**250 – 250.93**	**Diabetes Mellitus**
**Ischemic Vascular Disease**	
**410 – 410.92**	**AMI**
**411 – 411.89**	**Post-MI**
**412**	**Old AMI**
**413 – 413.9**	**Angina Pectoris**
**414.0 – 414.07**	**CAD**
**414.2**	**Chronic Total Occlusion of Coronary Artery**
**414.8**	**Other Chronic Ischemic Heart Disease (IHD)**
**414.9**	**Chronic IHD**
**429.2**	**Cardiovascular (CV) disease, unspecified**
**433 – 433.91**	**Occlusion and stenosis of pre-cerebral arteries**
**434 – 434.91**	**Occlusion of cerebral arteries**
**440.1**	**Atherosclerosis of renal artery**
**440.2 – 440.29**	**Atherosclerosis of native arteries of the extremities, unspecified**
**440.4**	**Chronic Total Occlusion of Artery of the Extremities**
**444 – 444.9**	**Arterial embolism and thrombosis**
**445 - 445.8**	**Atheroembolism**

### Measurements

MNCM compares practices on “optimal care” using a composite “all-or-none” measure that includes a mixture of process and outcome components. Diabetes requires values at goal for glucose control using Hemoglobin A1c (HbA1c) measurement, blood pressure control using Blood Pressure (BP) measurement, and lipid control using Low Density Lipoprotein (LDL) measurement, documentation of non-smoking status and documentation of daily aspirin use. Vascular care requires values at goal for BP, LDL, documentation of non-smoking status and documentation of daily aspirin use. For this study we restricted our assessment to the outcome components of the composite measure: the three physiological parameters (HbA1c, BP, and LDL) for diabetes and the two physiological parameters (BP and LDL) for ischemic vascular disease. For public reporting, these measures are electronically extracted. This eliminated the need for extensive manual chart review. Aspirin (ASA) use and non-smoking status documentations have high compliance rates in Minnesota suggesting these two parameters add only a small portion to the overall optimal care metric [2008 Minnesota statewide average at goal: BP (56%), LDL (58%), HbA1c (58%) vs. Tobacco (83%), ASA (89%) for diabetes; and BP (58%), LDL (65%) vs. Tobacco (82%), ASA (93%) for IVD] [16]. In addition, being process measures, these assessments are not expected to differ between short-term and long-term patients. We defined our modified “optimal care” measure for diabetes as a patient whose most recent assessment during the measurement period (2008) was at goal for HbA1c (goal < 7.0% [53 mmol/mol]), LDL (goal < 100 mg/dl), and blood pressure (goal < 130/80 mmHg). Our “optimal care” measure for IVD was defined as a patient whose most recent assessment during 2008 was at goal for blood pressure (goal < 130/80 mmHg) and during the period of 10/1/2007 to 12/31/2008 was at goal for LDL (goal < 100 mg/dl) [MNCM had allowed 5 quarters of data for LDL]. If values were not recorded during the measurement time frame, these were considered missing and not in control (MNCM defines missing values as a failure in performance).

### Statistical analysis

For both specialty care and primary care: patients with short and long term care were compared within each disease cohort with two sample t-tests for continuous data and chi-square analysis for categorical data. Assessment of differences on “optimal care” measures between short and long term care measures were based on logistic regression models incorporating age and sex of patients.

## Results

3214 diabetic and 1027 vascular patients were attributed to primary care providers. Patients who met the visit criteria for inclusion in both primary care and specialty care were attributed only to primary care (n = 62 diabetic and n = 60 vascular patients). 2701 diabetic and 1759 vascular patients were attributed to specialist providers, endocrinology and cardiology, respectively.

For specialty care and primary care, the characteristics comparing those with short-term care (<90 days) to those with long-term care (>90 days) are displayed in Tables 2 and 3 for diabetes and IVD, respectively. Specialists saw significantly higher proportions of short-term patients with diabetes (15.4% vs. 1.9%, p < 0.001) and patients with IVD (29.2% vs. 3.2%, p < 0.001) than primary care providers. Missing laboratory values were more common in short-term care patients than long-term patients (diabetes: 21.1% vs. 9.4%, p < 0.001; IVD: 9.7% vs. 7.1%, p = 0.036) resulting in more missing data for the specialist than the primary care providers. Furthermore, the percentage of patients at “optimal care” was statistically lower for those with short-term care than those with long-term care [diabetes: 7.5% versus 23.7% (p < 0.001); IVD: 41.0% versus 52.2% (p < 0.001)]. After adjustment for patient age and sex, patients with short-term care are 4.6 times less likely to meet diabetes optimal care measures and 2.5 times less likely to meet IVD optimal care measures. Short-term care patients seeing specialists are also significantly less likely to be from the nearby geographic area (within 120 miles) for diabetes [32.6% versus 67.5% (p < 0.001)] and for IVD [46.2% versus 56.3% (p < 0.001), reflecting the nature of our national referral practice.

**Table 2:** **Diabetes – short versus long term care**

**Characteristics**	**Endocrinology**				**Primary care**			
	**≤90 days of care**	**>90 days of care**	**p-value**	**Combined overall**	**≤90 days of care**	**>90 days of care**	**p-value**	**Combined overall**
N, (%)	417 (15.4%)	2284 (84.6%)		2701	62 (1.9%)	3152 (98.1%)		3214
Male, %	58.8	55.7	0.240	56.2	56.5	54.3	0.734	54.3
Age, Mean (SD)	54.77 (13.26)	57.22 (12.74)	<0.001	56.8 (12.8)	51.7 (12.50)	58.03 (11.07)	<0.001	57.9 (11.1)
Within 120 miles, %	32.6	67.5	<0.001	62.1	91.9	98.4	<0.001	98.3
MN Resident, %	29.5	63.8	<0.001	58.5	91.9	98.2	<0.001	98.1
SE MN Resident, %	15.1	45.1	<0.001	40.5	91.9	96.6	0.049	96.5
Missing Data, %	22.1	12.2	<0.001	13.7	14.5	7.4	0.035	7.5
Optimal Diabetes Care, %	7.2	19.7	<0.001	17.8	9.7	18.8	0.068	18.6

**Table 3:** **Ischemic vascular disease – short versus long follow-up**

**Characteristics**	**Cardiology**				**Primary care**			
	**≤90 days of care**	**>90 days of care**	**p-value**	**Combined overall**	**≤90 days of care**	**>90 days of care**	**p-value**	**Combined overall**
N, (%)	513 (29.2%)	1246 (70.8%)		1759	33 (3.2%)	994 (96.8%)		1027
Male, %	76.2	71.6	0.047	72.9	66.7	74.0	0.343	73.8
Age, Mean (SD)	61.37 (9.48)	63.25 (8.59)	<0.001	62.7 (8.85)	60.24 (9.45)	62.88 (8.79)	0.091	62.8 (8.81)
Within 120 miles, %	46.2	56.3	<0.001	53.4	90.9	97.2	0.038	97.0
MN Resident, %	45.2	52.9	0.004	50.7	90.9	97.4	0.027	97.2
SE MN Resident, %	23.2	32.7	<0.001	29.9	90.9	95.2	0.268	95.1
Missing Data, %	10.1	7.6	0.084	8.3	3.0	6.3	0.439	6.2
Optimal IVD Care, %	41.3	53.1	<0.001	49.7	36.4	51.0	0.098	50.5

**Figure 1:**
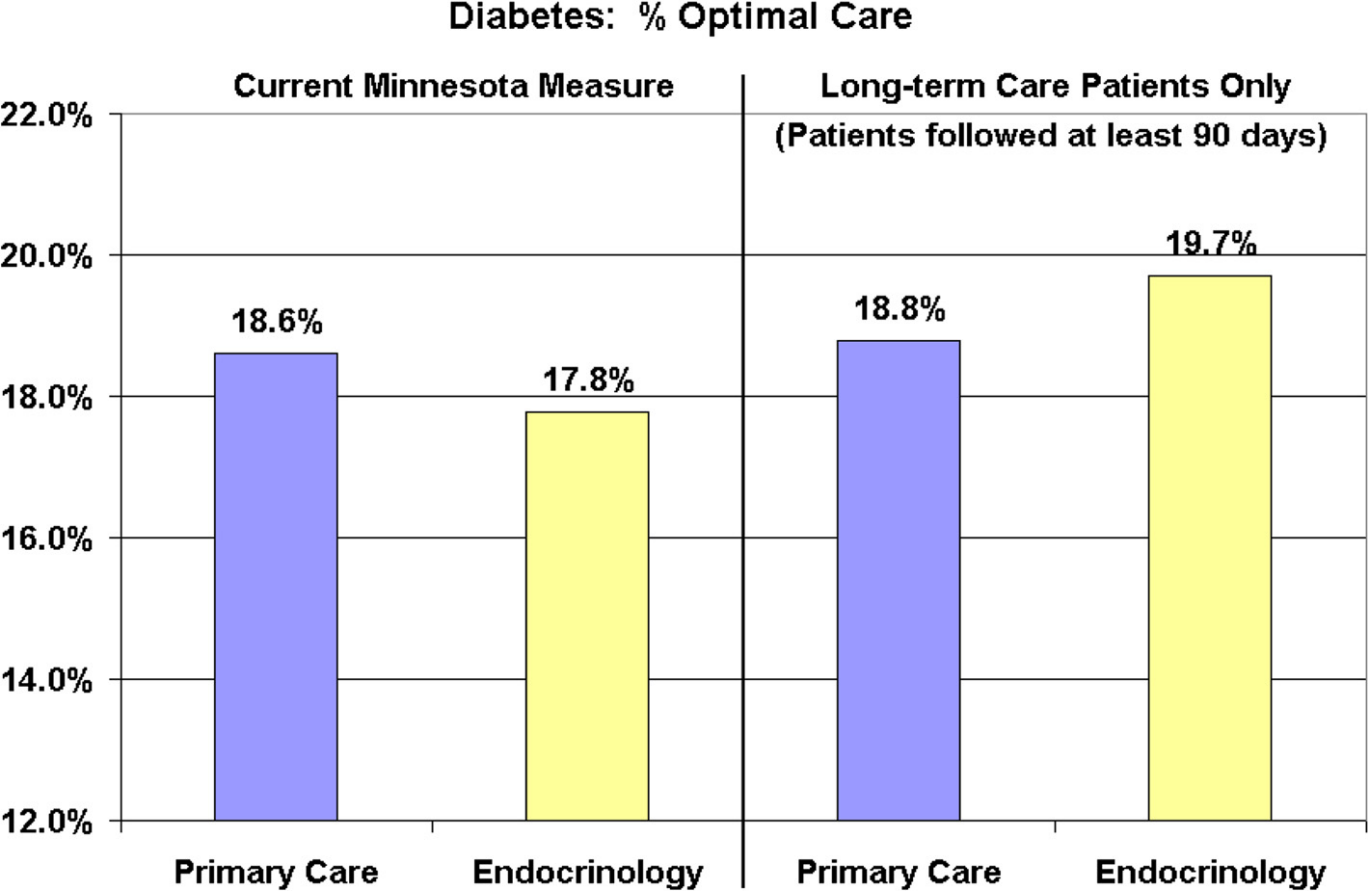
**Diabetes performance – primary care vs. specialty.**

Figure 1 shows the impact of including short-term patients among the diabetes cohort on the comparison of the performance of endocrinology to the performance of primary care. As currently defined by MNCM (both those with short and long term care), endocrinology had a lower percentage of patients at “optimal care” than did primary care. However, when restricting the measurement to patients with long-term care only (e.g., continuing care patients), endocrinology had a higher percentage of patients at “optimal care” than did primary care.

**Figure 2:**
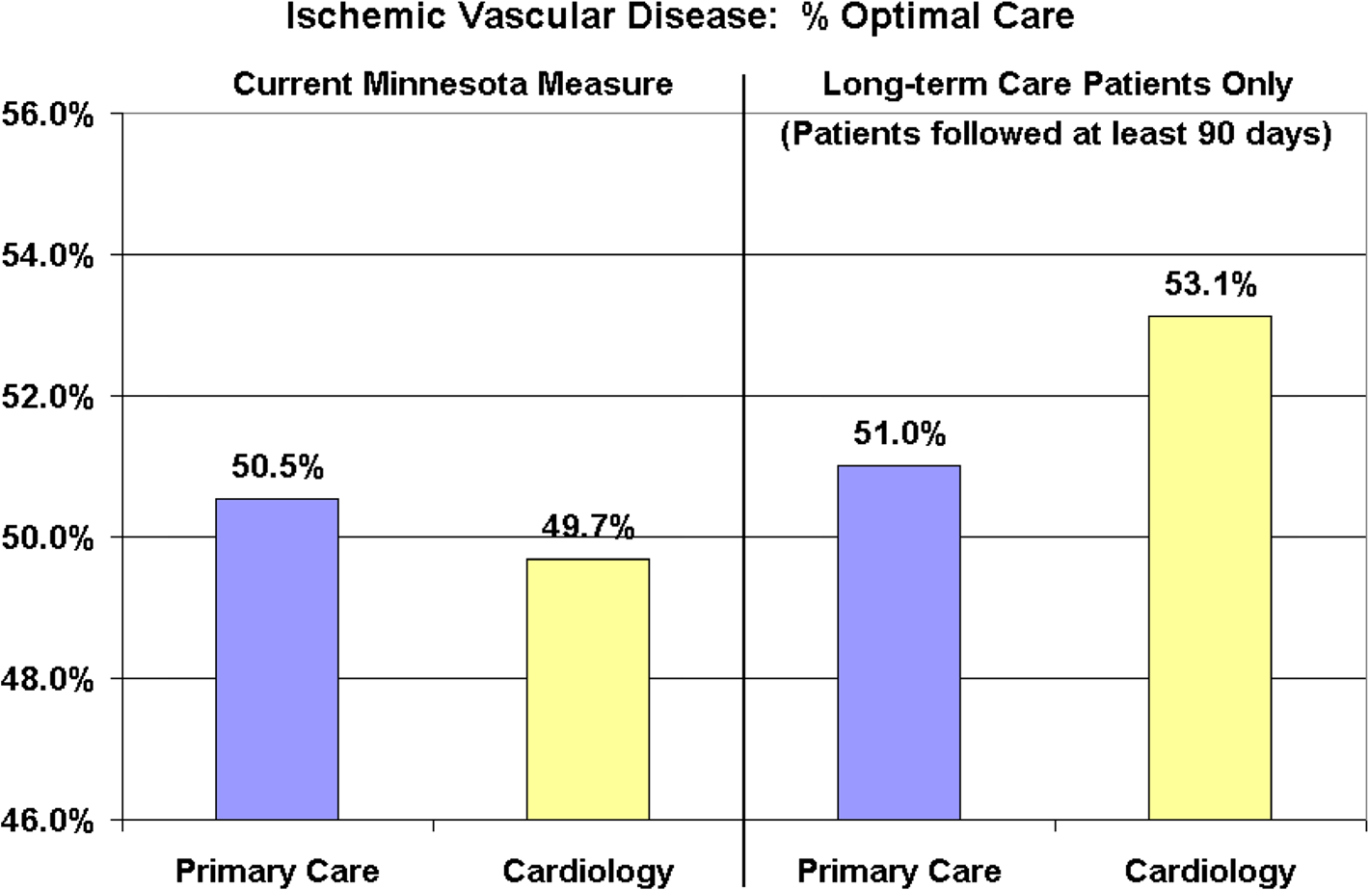
**Ischemic vascular disease performance – primary care vs. specialty.**

Figure 2 shows the similar impact of the definition of these patients on the performance of cardiology to the performance of primary care. Using the Minnesota measure including all patients in the denominator (both those with short and long term care), cardiology had a lower percentage of patients at “optimal care” than did primary care. However, when restricting the measurement to patients with long-term care only (continuing care patients), cardiology had a higher percentage of patients at “optimal care” than did primary care.

## Discussion

Using definitions currently used for public reporting, we compared a modified “optimal care” quality metric between primary care and specialty practice for two disease cohorts, based on time between each patient’s first and last visit during the study time frame. The measures reported in the Minnesota Quality Report [17] to assess the performance of care for diabetes and ischemic vascular disease consist of outcome metrics that clinically reflect care delivered over the long-term, and not care delivered over the short-term. However, these health outcome measures include both short and long term care patients in the measurement cohort. We showed that specialists have a significantly higher percentage of patients receiving short-term care. We also showed patients receiving short–term care have a significantly lower percentage at “optimal care”. When combining patients with short and long-term care, the percentage of patients at “optimal care” for the specialist was lower when compared to primary care. However, when comparing only patients with long-term care, the percentage of patients at “optimal care” was actually higher for the specialist when compared to primary care. Our findings are similar to specialty clinics throughout Minnesota where specialty clinics rank lower than primary care clinics with regards to performance according to the specified MNCM measurements.

Why were there fewer patients at “optimal” care in the short-term care patient pool? As outlined above, many had been referred to the specialist because of their poor control or complexity. Additionally, patients may have had laboratory measures performed at home just prior to the measurement period and not repeated at the referral site within the measurement period, due to their intended return to their local provider. Laboratory values reported from outside sources, while used to make clinical decisions, are often not captured in discrete fields in our electronic record and therefore are considered missing laboratory results (counted as a failure) for assessment purposes. Furthermore, short-term patients had insufficient follow-up time for A1C measures to reflect any treatment recommendations following their specialty visits.

Based on our analysis, a more equitable comparison between specialty practice and primary care would be to compare the patients they are caring for over the long-term. Restricting the measured population to longer-term patients could be easily attained by modifying the denominator definition to require two visits within two years to be at least 90 days apart. Applied across all clinics, this would reduce the demonstrated bias for specialty practice performance metrics, with minimal impact on primary care performance. Alternatively, one could compare cohorts of patients seen within similar settings – specialty care to specialty care and primary care to primary care. Finally, one could eliminate public reporting all together and use the data for internal quality improvement only.

Our study was limited by several factors. It was performed at only one institution, an academic center with a geographically large referral practice. Though similar results may be expected at other specialty practices, the magnitude of the difference between referred (short-term) and continuity (long-term) patients will depend on the mix of these patients and may be smaller, especially at community specialty practices. We also were not able to determine whether most of the differences between short-term and long-term patients were due to initial disease severity, other differences between the patients, or treatment differences. However, due to the short exposure of these chronically ill patients to the specialty provider, we feel that this issue does not impact our conclusions. As in MNCM reporting, our study relied only on data available by the reporting system to the clinicians, and no active follow-up was used to obtain more recent laboratory or blood pressure results. Furthermore, the MNCM measurements may be subject to other criticisms (e.g., use of the last recorded measure to reflect chronic disease control, lack of adjustment for multiple chronic disease, treating missing laboratory values as lack of disease control). We were not able to address all of these issues in this study.

## Conclusions

Value in health care has been defined as the health outcomes achieved per dollar spent [18]. When comparing value across different providers, the health outcomes must be risk adjusted for the patient’s initial condition to prevent bias [19]. How the measured cohort is defined can also affect the perceived performance [9]. We have demonstrated that the perceived performance or public “rank” using current Minnesota State mandated quality metrics for the specialty practice can be impacted by the percent of short-term patients seen. Patients receiving short-term care that are included within the cohort of an outcome measure add an additional confounding factor that must be adjusted for when designing outcome measures. These results are important nationally as these same MNCM diabetes and vascular optimal care metrics have been endorsed by the National Quality Forum [20] and are being incorporated in the Medicare program Group Provider Quality Reporting System. Health care policy makers and public quality reporting initiatives must be aware that the same measure applied across different patient populations is not always equitable. As we move towards value based payment methods, appropriate adjustments must be applied to outcome measures and the population assessed in order to fairly reimburse providers across different types of practices. Physicians and clinics must be held accountable for the quality of their care, but only the care within their control.

## Abbreviations

ADA: American Diabetes Association
ASA: Aspirin
BP: Blood pressure
EMR: Electronic medical record
HbA1c: Hemoglobin A1c
IVD: Ischemic vascular disease
LDL: Low density lipoprotein
MNCM: Minnesota community measurement
